# Supplementary material for: Elevated extracellular matrix protein 1 in circulating extracellular vesicles supports breast cancer progression under obesity conditions
Source: Nat Commun. 2024 Feb 24;15:1685. doi: 10.1038/s41467-024-45995-5 (PMC10894219; doi:10.1038/s41467-024-45995-5)
Supplement: Supplementary file 1 — Supplementary Figs. [file 41467_2024_45995_MOESM1_ESM.pdf]

Supplementary Figure S1

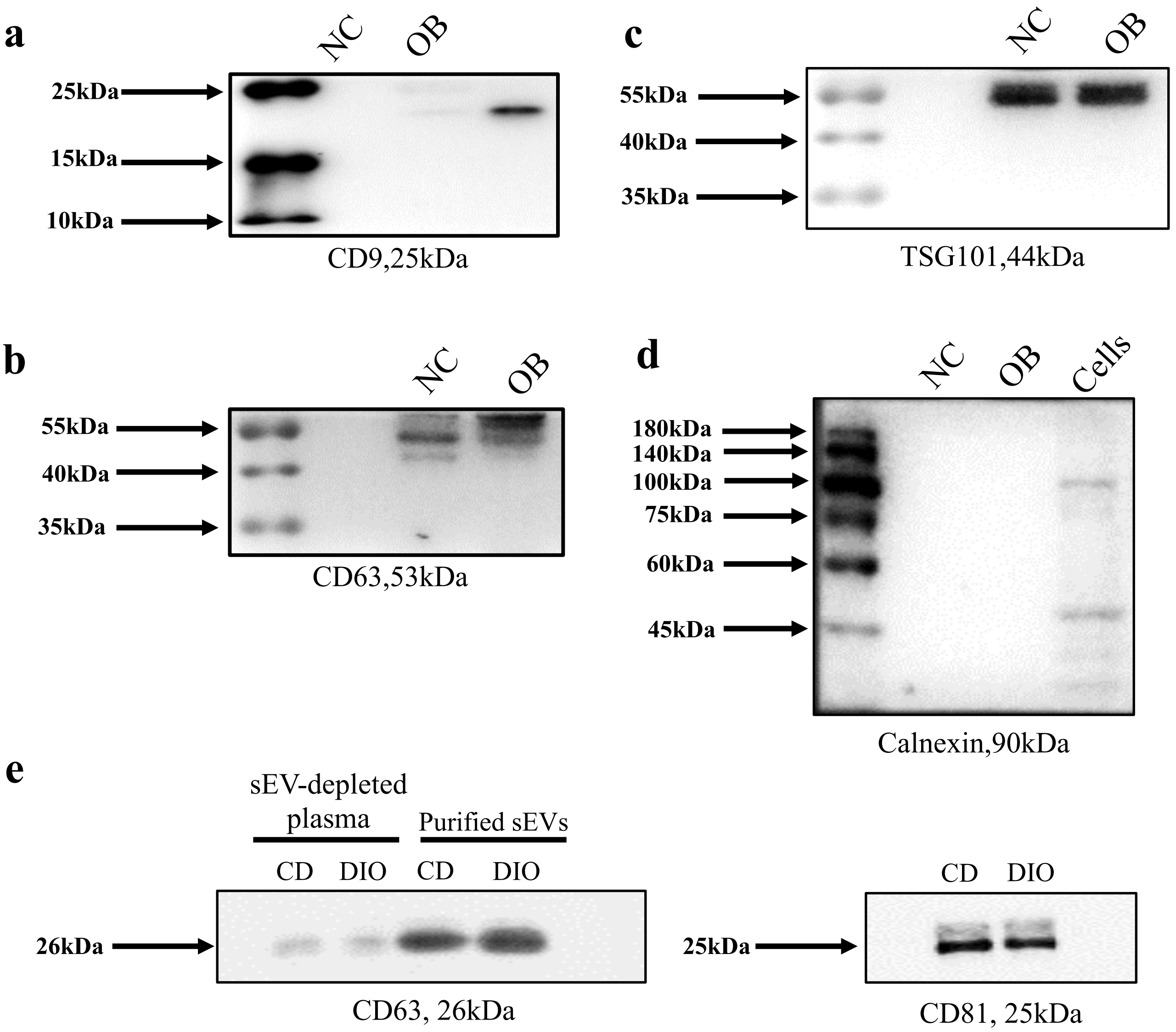

Supplementary Figure S1

Representative Western blots showing the expressions of sEV markers

sEV markers in the circulating sEVs purified from the plasma of human subjects **(a)** CD9, **(b)** CD63, **(c)** TSG101, **(d)** calnexin. **(e)** Expressions of sEV markers CD63 and CD81 in the circulating sEVs purified from the plasma of mouse models. NC, normal weight healthy human subjects; OB, human subjects with obesity or overweight; CD, control diet fed mice; DIO, high-fat diet-induced obesity mice; cells, cell sample is included as positive control.

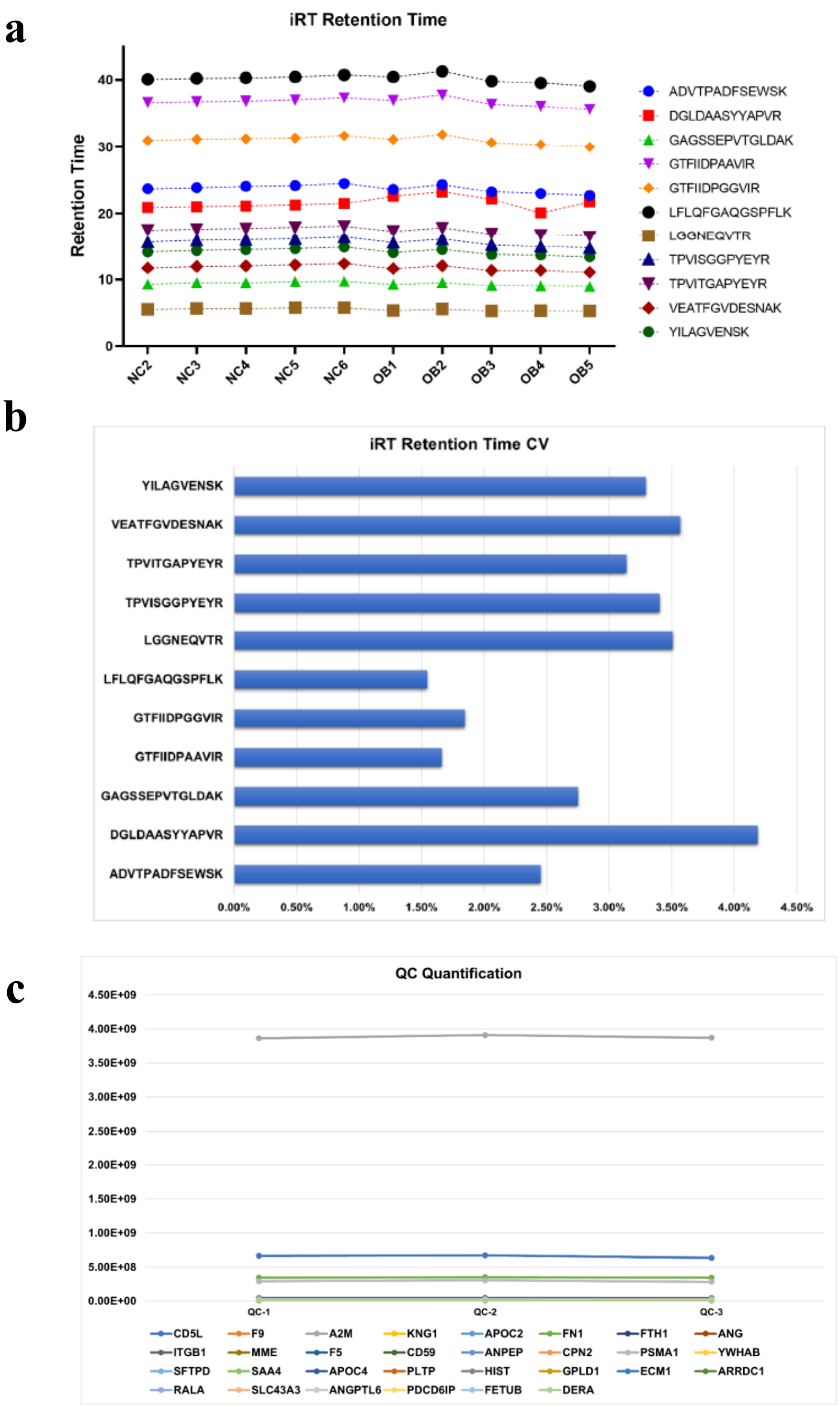

Supplementary Figure S2

Quality control tests for MRM-mass spectrometry

(a) iRT retention time and **(b)** iRT retention time CV of the sample peptide in the MRM-mass spectrometry that indicate the qualitative stability of the repeated tests and small systemic errors of the samples. **(c)** The quantitative results of the detected proteins in the quality control (QC) samples.

Supplementary Figure S3

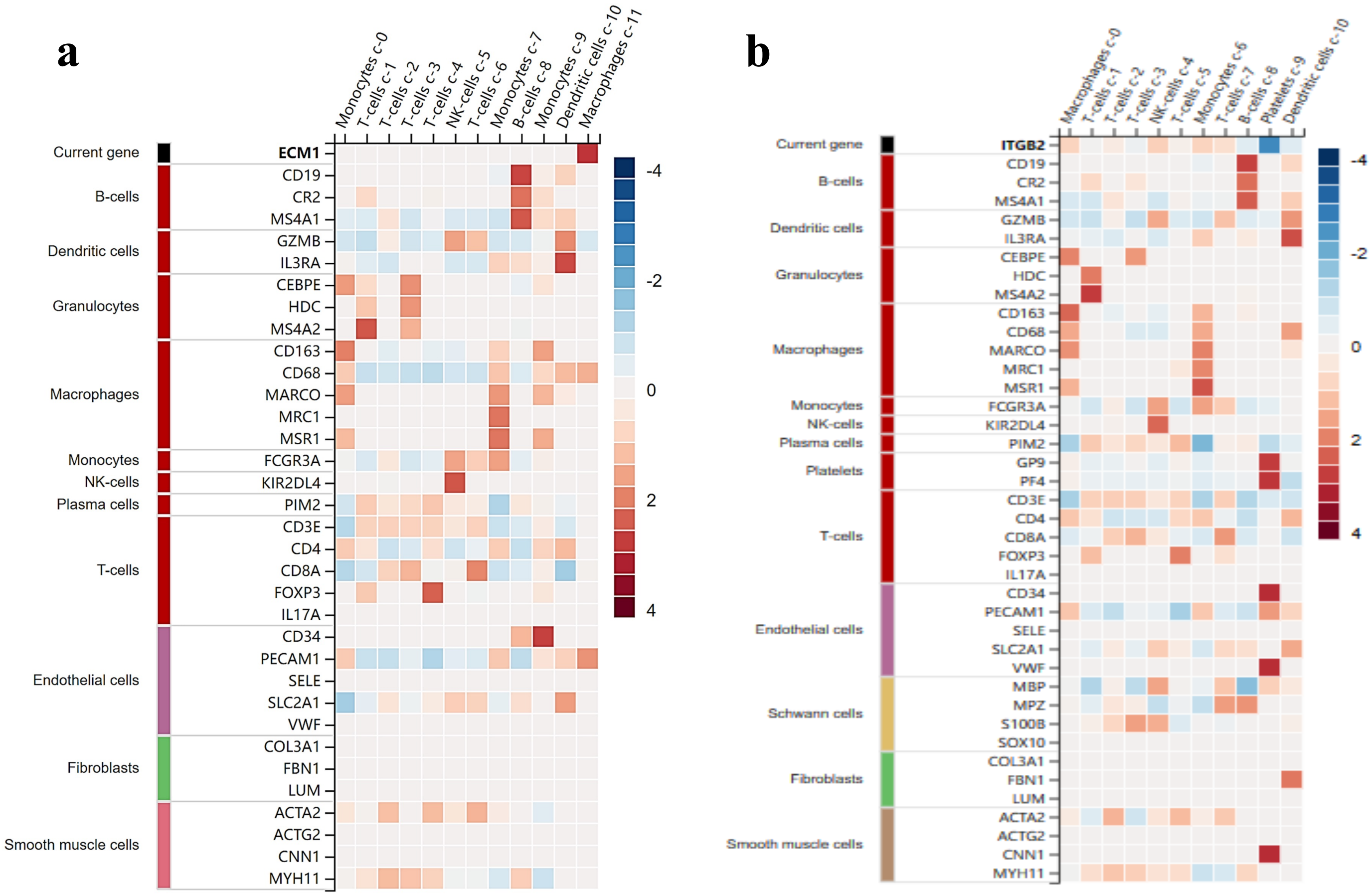

Supplementary Figure S3
Expressions of EMC1 and ITGB2 in macrophages
Single cell sequencing analysis showing **(a)** ECM1 expressions in peripheral blood mononuclear cells (PBMC) including macrophages, **(b)** ITGB2 (integrin-β2) expressions in PBMC including macrophages.

Supplementary Figure S4

a

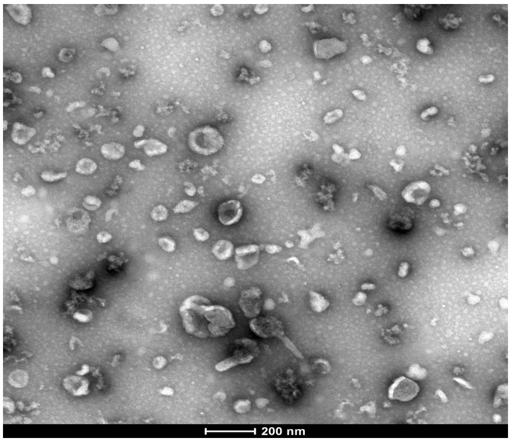

b

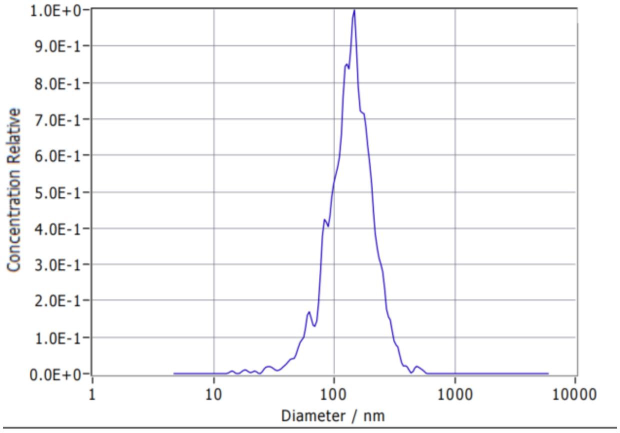

Supplementary Figure S4
sEVs released by RAW264.7 cells
(a) Transmission electron microscopy (TEM) and **(b)** nanoparticle tracking analysis (NTA) of the sEVs released by RAW264.7 cells.

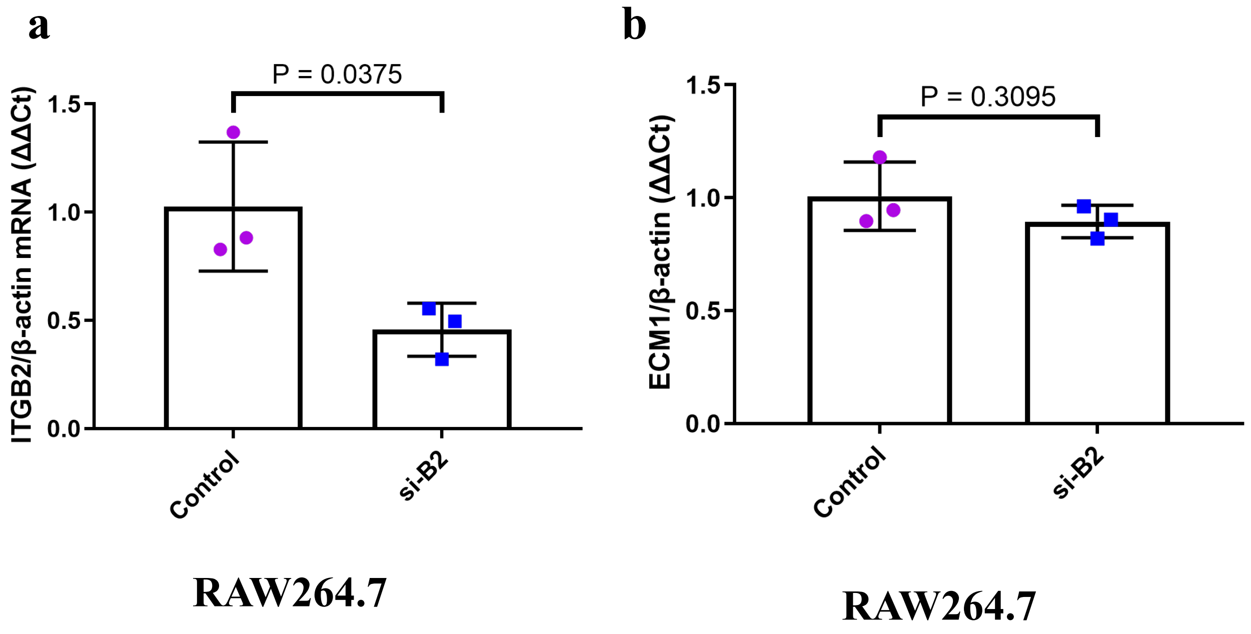

Supplementary Figure S5

ITGB2 and ECM1 mRNA levels in integrin-β2-knockdown and control RAW264.7 cells

(a) ITGB2 and **(b)** ECM1 mRNA levels in the integrin-β2-knockdown and control RAW264.7 cells. n=3 individual experiments; two-sided unpaired *t*-test for (**a, b**); p values are indicated in graphs. si-β2, integrin-β2-knockdown cells; NC, control cells. Source data are provided as a Source Data file.

Supplementary Figure S6

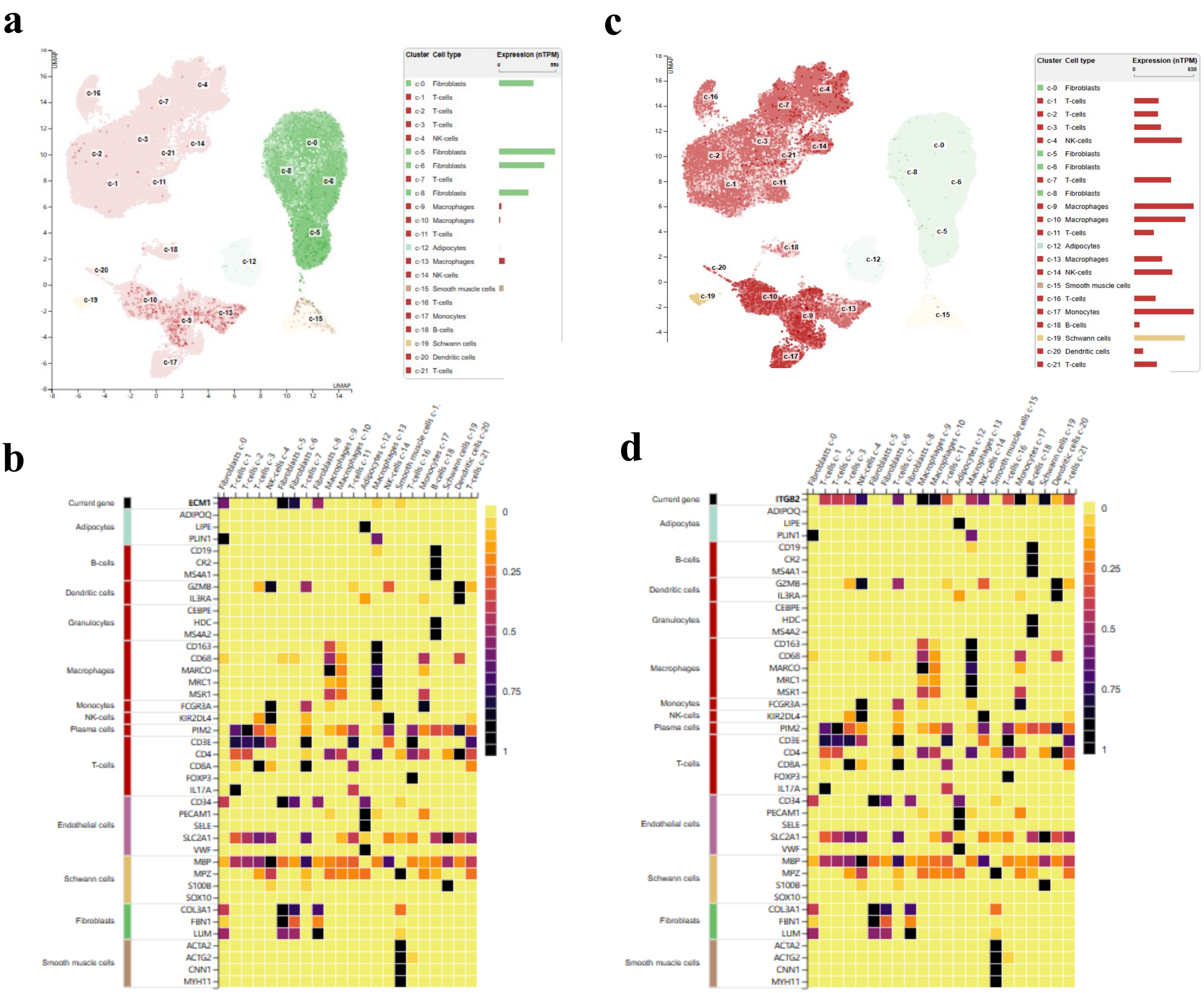

Supplementary Figure S6

Expressions of ECM1 and integrin-β2 in adipose tissues

Single cell sequencing analysis showing **(a-b)** ECM1 expression in adipose tissues, **(c-d)** integrin-β2 expression in adipose tissues.

Supplementary Figure S7

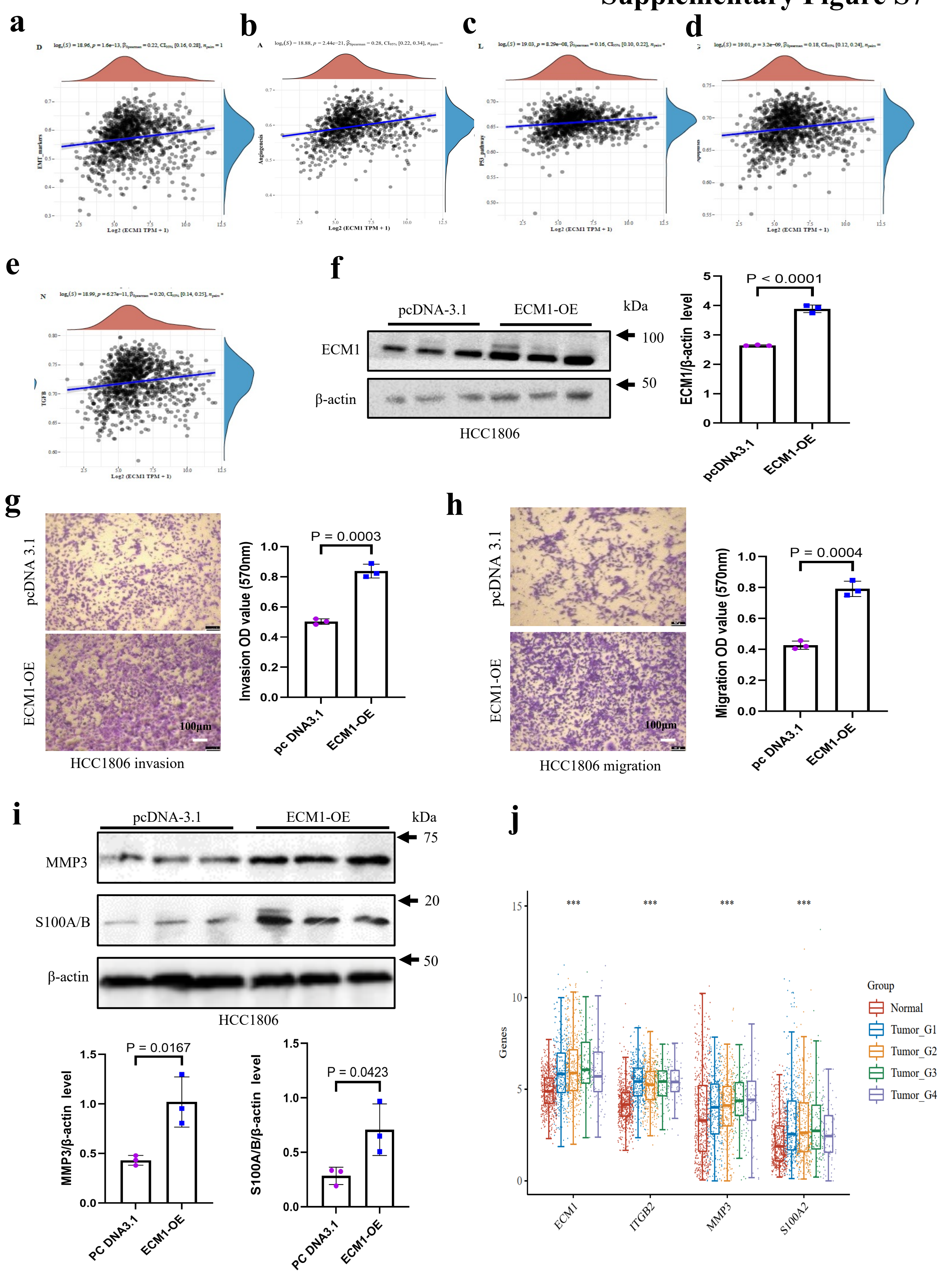

Supplementary Figure S7

Overexpression of ECM1 increases MMP3 and S100A/B expressions and metastatic potential of BC cells

Virtual Flow analysis showing the correlation between ECM1 and oncogenic signaling pathway scores in human breast cancer **(a)** epithelial-mesenchymal-transition (EMT) markers, **(b)** angiogenesis, **(c)** P53, **(d)** apoptosis and **(e)** transforming growth factor beta (TGF-beta). **(f)** ECM1 protein levels in ECM1-overexpressed (ECM1-OE) and empty vector (pcDNA-3.1)-transfected human breast cancer cells HCC1806. **(g)** Invasion and **(h)** migration of the ECM1-overexpressed and control HCC1806 cells. **(i)** Expressions of MMP3 and S100A/B in the ECM1-overexpressed and empty vector-transfected HCC1806 cells. **(j)** Prognosis analysis showing the correlations between the expressions of ECM1, MMP3, VEGF and S100A2 in 898 breast cancer patients who were divided into 4 stages, including G 1(N=333), G 2 (N=366), G 3 (N=120), G 4 (N=79). A total of 572 normal tissues (N=113 para-cancer tissue from TCGA database; N=459 from Genotype-Tissue Expression database) were included as controls. MMP3, matrix metallopeptidase 3; VEGF, vascular endothelial growth factor; ECM1, extracellular matrix protein 1. Shown is the mean ± SD; n = 3 independent experiments; two-sided unpaired *t*-test for (**f-i**); The Kruskal-Wallis test with the Wilcoxon's multiple comparison for (j); p values are indicated in graphs. Source data are provided as a Source Data file.

Supplementary Figure S8

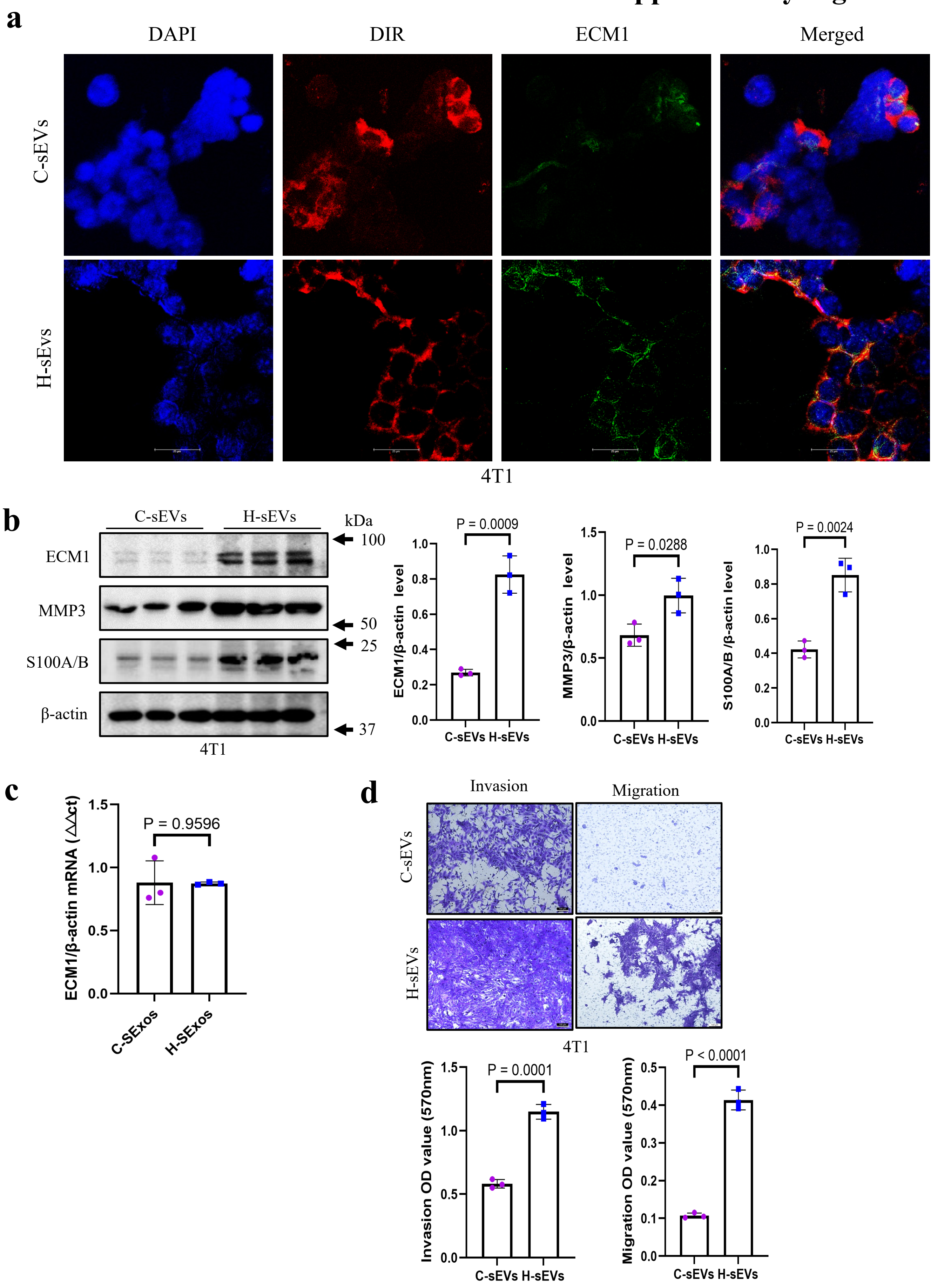

Supplementary Figure S8
sEVs treatments in 4T1 cells
(a) Expression of ECM1 protein in sEVs-treated 4T1 cells shown by immunofluorescence staining. (**b)** Expressions of ECM1, MMP3 and S100A/B proteins in sEVs-treated 4T1 cells. **(c)** Expression of ECM1 mRNA levels in the sEVs-treated 4T1 cells. **(d)** Invasion and migration of sEVs-treated 4T1 cells. Shown is the mean ± SD; n = 3 independent experiments; two-sided unpaired *t*-test for (**a-d**); p values are indicated in graphs. C-sEVs, circulating sEVs in CD mice; H-sEVs, circulating sEVs in high fat diet-feeding mice; ECM1, extracellular matrix protein 1; MMP3, matrix metallopeptidase 3. Source data are provided as a Source Data file.

Supplementary Figure S9

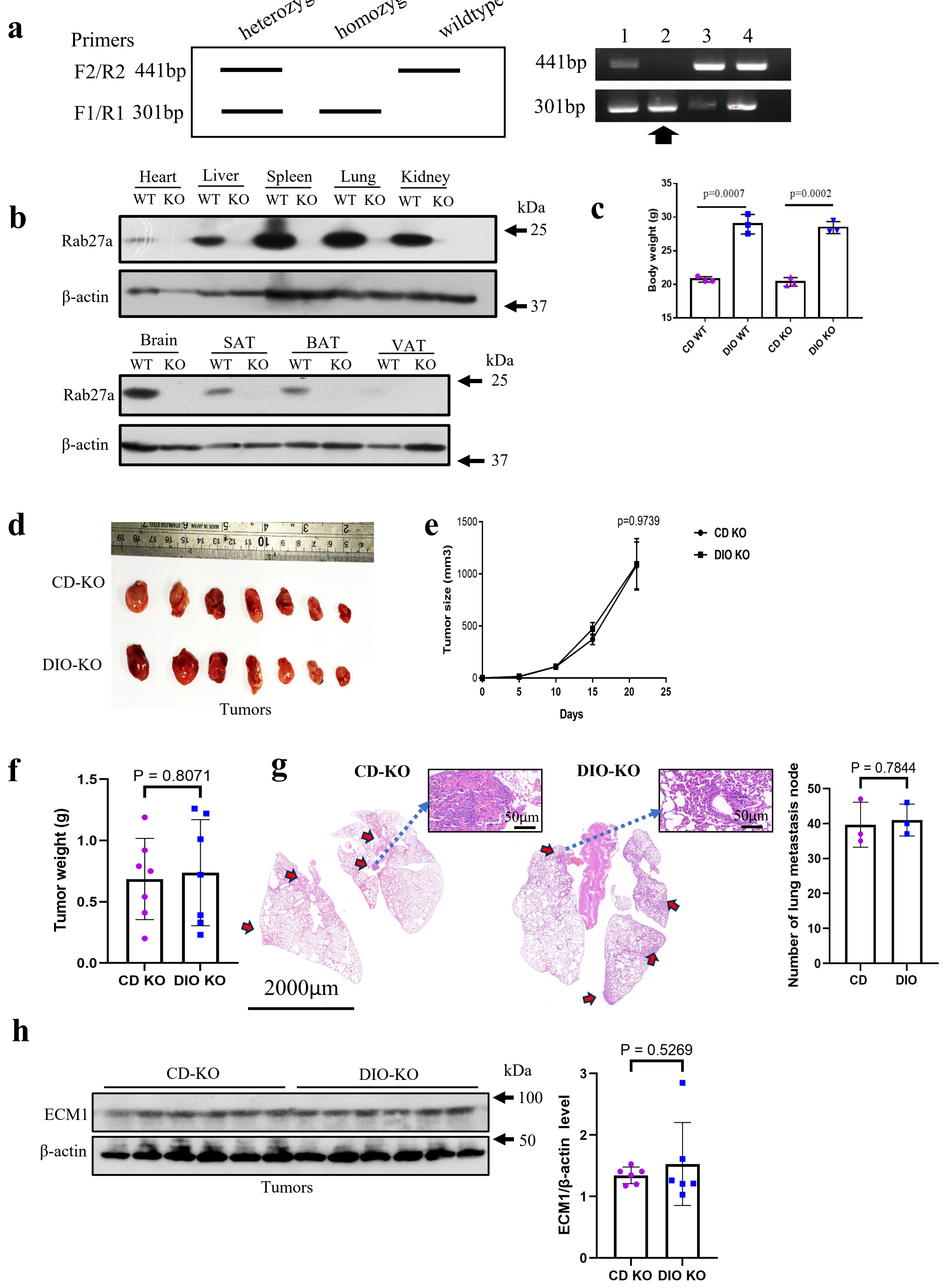

Supplementary Figure S9

Obesity fails to increase breast cancer growth and metastasis in Rab27a knockout mouse model

(a) Genotyping of B6/J-Rab27a-Cas9-KO mice and **(b)** Rab27a protein expressions in the heart, liver, spleen, lung, kidney, brain, subcutaneous adipose tissue (SAT), epididymal adipose tissues (EAT) and visceral adipose tissue (VAT) of the B6/J-Rab27a-Cas9-KO (KO) mice. **(c)** Establish DIO mouse model with the B6/J-Rab27a-Cas9-KO mice and isogenic mice. **(d)** Tumors, **(e)** tumor size, **(f)** tumor weight, **(g)** lung metastasis and **(h)** tumor ECM1 protein levels in the mouse models. Mouse tumor sizes are presented as the mean ± SEM; other data are presented as mean ± SD; two-sided unpaired *t*-test for (**d-h**); n = 7 mice in each group; p values are indicated in graphs. WT, wildtype; KO, B6/J-Rab27a-Cas9-KO mice; CD-KO, control diet fed B6/J-Rab27a-Cas9-KO mice; DIO-KO, high fat diet induced obesity B6/J-Rab27a-Cas9-KO mice; ECM1, extracellular matrix protein 1. Source data are provided as a Source Data file.

Supplementary Figure S10

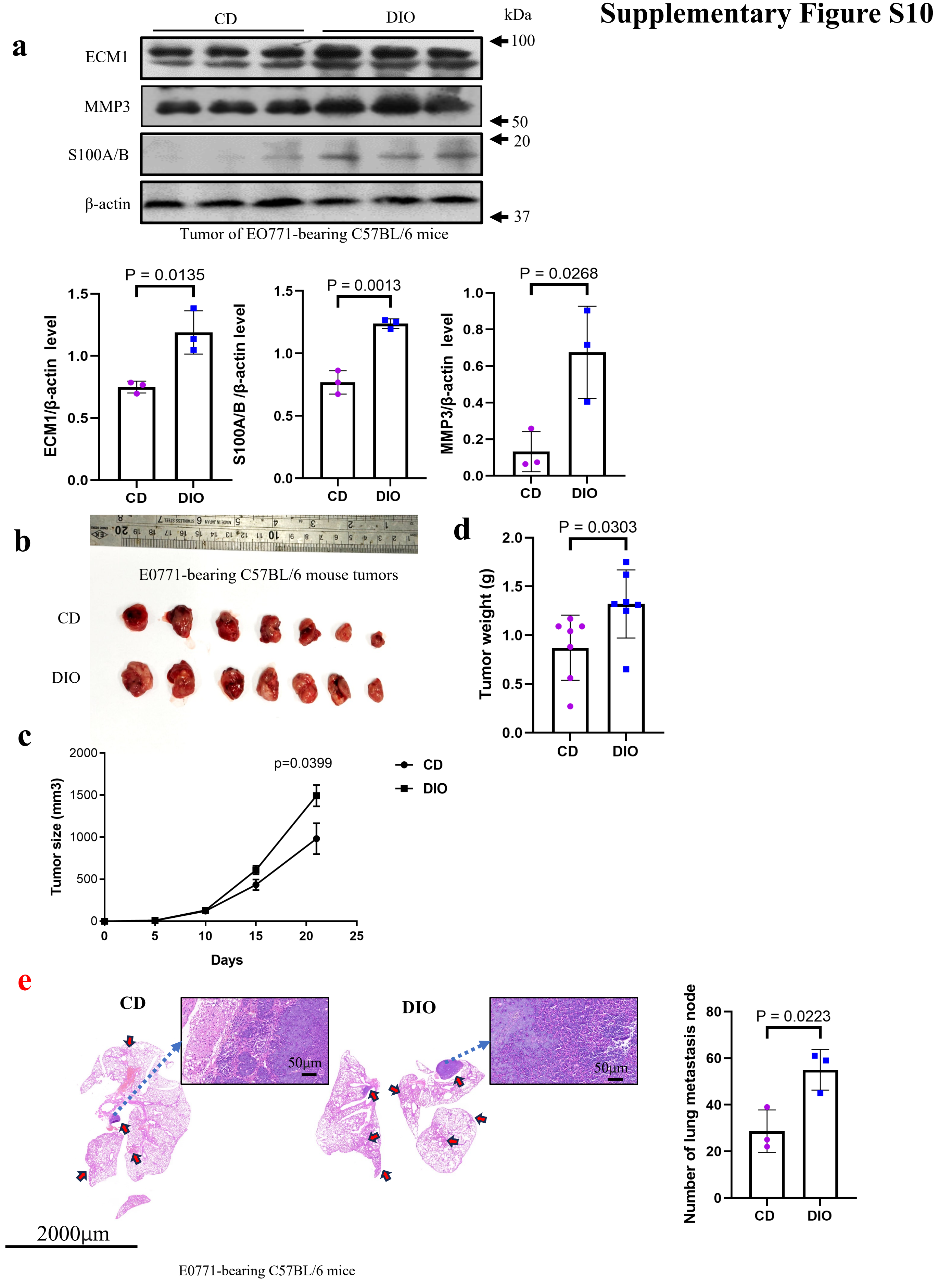

Supplementary Figure S10

Obesity increases breast cancer growth and metastasis in E0771-bearing C57BL/6 mice

(a) Protein expressions of ECM1, MMP3 and S100A/B in the tumors of the E0771-bearing CD and DIO mice. **(b)** Tumors, **(c)** tumor size, **(d)** tumor weight and **(e)** lung metastasis of the E0771-bearing CD and DIO mice. Mouse tumor sizes are presented as the mean ± SEM, other data are presented as mean ± SD; all two-sided unpaired *t*-test; n = 7 mice in each group, p values are indicated in graphs. ECM1, extracellular matrix protein 1; MMP3, matrix metallopeptidase 3; CD, control diet; DIO, high fat diet induced obesity. Source data are provided as a Source Data file.

Supplementary Figure S11

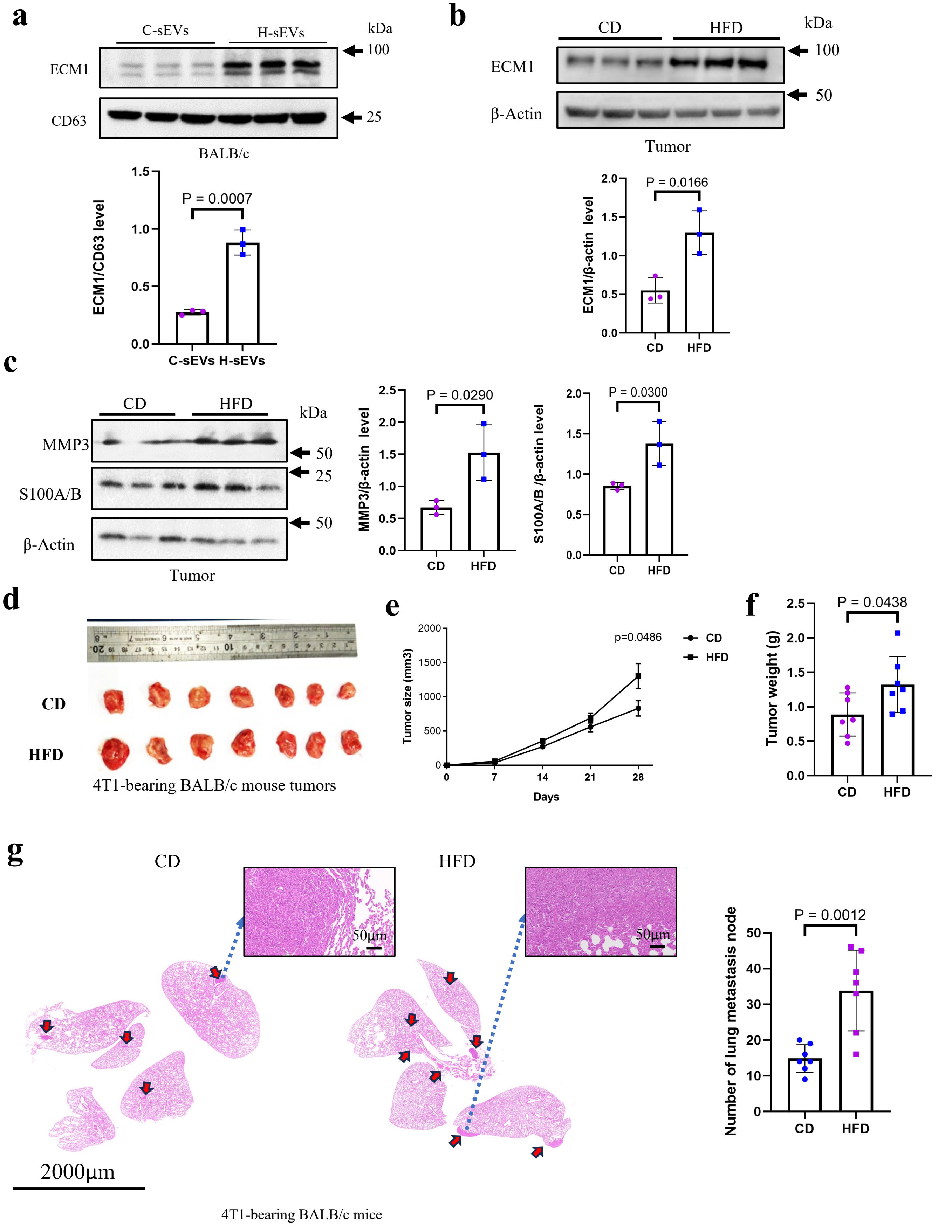

Supplementary Figure S11

Obesity increases breast cancer growth and metastasis in 4T1-bearing BALB/c mice

(a) ECM1 protein levels in the circulating sEVs of CD mice (C-sEVs) and HFD mice (H- sEVs). Protein expressions of **(b)** ECM1 and **(c)** MMP3 and S100A/B in the tumors of the 4T1-bearing BALB/c mice after dietary intervention. **(d)** Tumors, **(e)** tumor size, **(f)** tumor weight and **(g)** lung metastasis of the 4T1-bearing BALB/c mice after dietary intervention. Mouse tumor sizes are presented as the mean ± SEM; other data are presented as mean ± SD; all two-sided unpaired *t*-test; n = 7 mice in each group, p values are indicated in graphs. ECM1, extracellular matrix protein 1; MMP3, matrix metallopeptidase 3; CD, control diet; HFD, high fat diet. Source data are provided as a Source Data file.

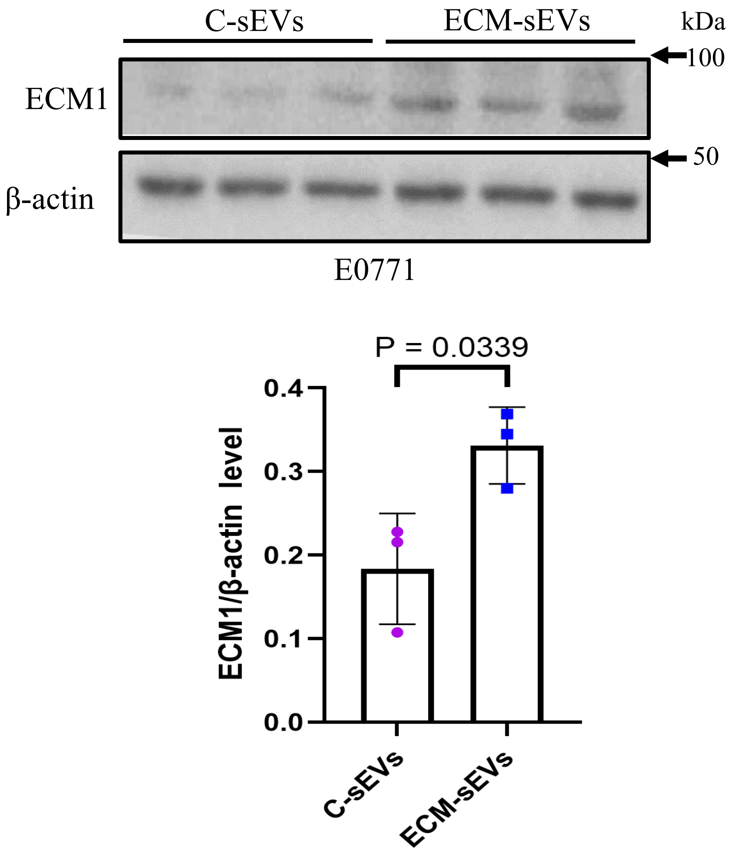

Supplementary Figure S12
Treatments with ECM1 construct-loaded sEVs increase ECM1 protein levels in the breast cancer cells
ECM1 protein levels in HCC1806 cells after receiving ECM1-construct-loaded sEVs treatments. Shown is mean ± SD; n = 3 individual experiments; two-sided unpaired *t*-test; p values are indicated in graphs. C-sEVs, circulating sEVs in CD-fed C57BL/6 mice; ECM-sEVs, ECM1 construct-loaded C-sEVs; ECM1, extracellular matrix protein 1. Source data are provided as a Source Data file.
