# Supplementary material for: Elevated extracellular matrix protein 1 in circulating extracellular vesicles supports breast cancer progression under obesity conditions
Source: Nat Commun. 2024 Feb 24;15:1685. doi: 10.1038/s41467-024-45995-5 (PMC10894219; doi:10.1038/s41467-024-45995-5)
Supplement: Supplementary file 3 — Reporting Summary [file 41467_2024_45995_MOESM3_ESM.pdf]

nature portfolio

Corresponding author(s): Jianfeng Bao, Aiping Lyu, Hiu Yee Kwan

Last updated by author(s): Dec 20, 2023

Reporting Summary

Nature Portfolio wishes to improve the reproducibility of the work that we publish. This form provides structure for consistency and transparency in reporting. For further information on Nature Portfolio policies, see our Editorial Policies and the Editorial Policy Checklist.

Statistics

For all statistical analyses, confirm that the following items are present in the figure legend, table legend, main text, or Methods section.

n/a	Confirmed	
☐	☒	The exact sample size (*n*) for each experimental group/condition, given as a discrete number and unit of measurement
☐	☒	A statement on whether measurements were taken from distinct samples or whether the same sample was measured repeatedly
☐	☒	The statistical test(s) used AND whether they are one- or two-sided<br>*Only common tests should be described solely by name; describe more complex techniques in the Methods section.*
☐	☒	A description of all covariates tested
☐	☒	A description of any assumptions or corrections, such as tests of normality and adjustment for multiple comparisons
☐	☒	A full description of the statistical parameters including central tendency (e.g. means) or other basic estimates (e.g. regression coefficient) AND variation (e.g. standard deviation) or associated estimates of uncertainty (e.g. confidence intervals)
☐	☒	For null hypothesis testing, the test statistic (e.g. *F*, *t*, *r*) with confidence intervals, effect sizes, degrees of freedom and *P* value noted<br>*Give P values as exact values whenever suitable.*
☒	☐	For Bayesian analysis, information on the choice of priors and Markov chain Monte Carlo settings
☒	☐	For hierarchical and complex designs, identification of the appropriate level for tests and full reporting of outcomes
☒	☐	Estimates of effect sizes (e.g. Cohen's *d*, Pearson's *r*), indicating how they were calculated

Our web collection on statistics for biologists contains articles on many of the points above.

Software and code

Policy information about availability of computer code

Data collection	qPCR analyses were performed by the ABI ViiA 7 real-time PCR system (Applied Biosystems).
Data analysis	GraphPad Prism V8 for Window OS was used for statistical analyses.<br>qPCR results were analyzed by ViiA 7 Real-time PCR system software (QuantStudio Software v1.6.1)<br>Confocal imaging was performed and analyzed by LAS X microscope software (version 3.7.4_23463)<br>Analysis of single cell data: R version 4.0.3<br>Database proteome discoverer 2.4 was used as a proteomic analysis software

For manuscripts utilizing custom algorithms or software that are central to the research but not yet described in published literature, software must be made available to editors and reviewers. We strongly encourage code deposition in a community repository (e.g. GitHub). See the Nature Portfolio guidelines for submitting code & software for further information.

Data

Policy information about availability of data

All manuscripts must include a data availability statement. This statement should provide the following information, where applicable:

- Accession codes, unique identifiers, or web links for publicly available datasets
- A description of any restrictions on data availability
- For clinical datasets or third party data, please ensure that the statement adheres to our policy

All relevant data supporting the key findings of this study are available within the article and its supplementary information files. The datasets analyzed during the current study are available in PRIDE under accession code PXD041236 and PXD041294, The Cancer Genome Atlas Program, Genptype-Tissue Expression database, and Human Protein Atlas. Source data are provided with this paper.

Human research participants

Policy information about studies involving human research participants and Sex and Gender in Research.

Reporting on sex and gender	11 males and 37 females in normal weight healthy control group; 77 males and 19 females in BMI group
Population characteristics	A total of 48 healthy people (11 males and 37 females, 34.19 ± 11.98 years old) with BMI ≤ 23 and without acute and chronic diseases were included in the normal weight healthy control group. A total of 96 people (77 males and 19 females, 37.19 ± 13.53 years old) with BMI ≥ 25 and without any infectious diseases, cancer, surgery, and genetic diseases were included in the obese group.
Recruitment	Patients recruited for the normal physical examination, without any patient selection, obtained informed consent from each individual at Hangzhou Xixi Hospital,Zhejiang province, China
Ethics oversight	The study protocol was conducted following the ethical guidelines of the hospital ethics committee, and the Zhejiang Provincial Health Committee approved the procedure. The protocol is in compliance with the Helsinki declaration.

Note that full information on the approval of the study protocol must also be provided in the manuscript.

Field-specific reporting

Please select the one below that is the best fit for your research. If you are not sure, read the appropriate sections before making your selection.

☒ Life sciences ☐ Behavioural & social sciences ☐ Ecological, evolutionary & environmental sciences

For a reference copy of the document with all sections, see nature.com/documents/nr-reporting-summary-flat.pdf

Life sciences study design

All studies must disclose on these points even when the disclosure is negative.

Sample size	Human: Group sample sizes was calculated at least 74 in the obese group and 37 in the healthy control group achieve 90.0% power a rate ratio of the group proportions of 1.67, allowing for a 10.0% loss.The proportion in the obese group is assumed to be 0.6 under the null hypothesis and 1.000 under the alternative hypothesis. The proportion in the thealthy control group is 0.6. The test statistic used is the two-side Z-Test with unpooled variance. The significance level of the test is 0.05.<br>Animal: We selected the sample size, based on our extensive experience with animal models and endpoints, with desirable significance (0.05) and power (>90%). We did not use animals that were not necessary.
Data exclusions	No data was excluded from the manuscript.
Replication	All of the experimental results were replicated as indicated in table and figure legends. Each experiment was independently repeated at least three times for in vitro studies. Only biological replicates were plotted and used for statistical analyses.
Randomization	Tissues from independently and randomly chosen mice at comparable developmental stages and sexes were collected for analyses and none of the samples was excluded. For the experiments involving transgenic animals, further allocations were based on the genotype of mice.<br>For the human samples, all samples were allocated according to the subtypes (e.g. healthy control vs overweight/obese subjects)
Blinding	Human subjects for proteomics were not blinded, due to the identification of obese or normal weight.<br>For molecular studies including western blotting and qPCR analyses, the experiments were not blinded, due to careful experimental setup and design.<br>Experiments of wild type mice of C57BL/6 and BALB/c were blinding. During the phenotyping experiments of transgenic mice, the experiments were performed blinded and the genotype was only disclosed after data analyses.<br>All mice histology scoring was performed by two blinded researchers.

Reporting for specific materials, systems and methods

We require information from authors about some types of materials, experimental systems and methods used in many studies. Here, indicate whether each material, system or method listed is relevant to your study. If you are not sure if a list item applies to your research, read the appropriate section before selecting a response.

Materials & experimental systems

n/a	Involved in the study
☐	☒ Antibodies
☐	☒ Eukaryotic cell lines
☒	☐ Palaeontology and archaeology
☐	☒ Animals and other organisms
☒	☐ Clinical data
☒	☐ Dual use research of concern

Methods

n/a	Involved in the study
☒	☐ ChIP-seq
☒	☐ Flow cytometry
☒	☐ MRI-based neuroimaging

Antibodies

Antibodies used: Anti-ECM1 (sc-365335, Santa Cruz, 1:1000), Anti-MMP3 (sc-21732, Santa Cruz, 1:600), Anti-S100a1 (nbp1-87103, Novus Biologicals, 1:1000), Anti-β-actin (sc-81178, Santa Cruz,1:5000), Rab27a (Abcam, 1:1000), CD63 (Abcam, 1:500),CD81(Santa Cruz, 1:1000).

Validation: All the validation and citation are available in the company websites, they are
Anti-ECM1 antibody (suitable for WB, IP, IF, IHC(P) ELISA; react with mouse, rat and human , https://www.scbt.com/p/ecm1-antibody-f-1;
Anti-MMP3 antibody (suitable for WB, IP, IF, IHC(P) ELISA; react with mouse, rat and human , https://www.scbt.com/p/mmp-3-antibody-1b4?requestFrom=search,
Anti-β-actin (suitable for WB, IP, IF, FM; react with mouse, rat and human?, https://www.scbt.com/p/beta-actin-antibody-actbd11b7?requestFrom=search,
https://www.scbt.com/p/cd81-antibody-b-11?requestFrom=search,
https://www.abcam.com/products/primary-antibodies/rab27a-antibody-1g7-ab55667.html,
https://www.abcam.com/products/primary-antibodies/cd63-antibody-late-endosome-marker-ab216130.html, Anti-S100A1 antibody (suitable for WB, IHC, IHCP react with mouse, rat and human , https://www.novusbio.com/products/s100a1-antibody_nbp1-87103.

Eukaryotic cell lines

Policy information about cell lines and Sex and Gender in Research

Cell line source(s): 4T1, E0771, HCC1806, RAW 264.7 cells were purchased from the American Type Culture Collection (ATCC, USA; CRL-2539 for 4T1, CRL-3461 for E0771, CRL-2335 for HCC1806, TIB-71 for RAW 264.7)

Authentication: All cell lines used are tested. The authentication was done by testing with the autosomal short tandem repeat (STR) loci with PowerPlex 16HS kit.

Mycoplasma contamination: The cells were tested negative for mycoplasma contamination

Commonly misidentified lines (See ICLAC register): No commonly misidentified cell lines were used

Palaeontology and Archaeology

Specimen provenance: *Provide provenance information for specimens and describe permits that were obtained for the work (including the name of the issuing authority, the date of issue, and any identifying information). Permits should encompass collection and, where applicable, export.*

Specimen deposition: *Indicate where the specimens have been deposited to permit free access by other researchers.*

Dating methods: *If new dates are provided, describe how they were obtained (e.g. collection, storage, sample pretreatment and measurement), where they were obtained (i.e. lab name), the calibration program and the protocol for quality assurance OR state that no new dates are provided.*

☐ Tick this box to confirm that the raw and calibrated dates are available in the paper or in Supplementary Information.

Ethics oversight: *Identify the organization(s) that approved or provided guidance on the study protocol, OR state that no ethical approval or guidance was required and explain why not.*

Note that full information on the approval of the study protocol must also be provided in the manuscript.

Animals and other research organisms

Policy information about studies involving animals; ARRIVE guidelines recommended for reporting animal research, and Sex and Gender in Research

Laboratory animals	Wild-type mice on C57BL/6J and BALB/c background of 6-8 weeks were from the Laboratory Animal Services Centre of The Chinese University of Hong Kong. Constitutive Rab27a knockout mice (B6/J-Rab27a-Cas9-KO) of 6-8 weeks were obtained by CRISPR/Cas9 technology (GemPharmatech, China).
Wild animals	No wild animals were used in this study.
Reporting on sex	Female
Field-collected samples	This study did not involve samples collected from the fields.
Ethics oversight	Animal experiments were conducted in accordance with the guidelines and with the approval of the Committees of Animal Ethics and Experimental Safety of Hong Kong Baptist University and procedures were approved by the Department of Health under Hong Kong legislation.

Note that full information on the approval of the study protocol must also be provided in the manuscript.

Dual use research of concern

Policy information about dual use research of concern

Hazards

Could the accidental, deliberate or reckless misuse of agents or technologies generated in the work, or the application of information presented in the manuscript, pose a threat to:

No	Yes	
☐	☐	Public health
☐	☐	National security
☐	☐	Crops and/or livestock
☐	☐	Ecosystems
☐	☐	Any other significant area

Experiments of concern

Does the work involve any of these experiments of concern:

No	Yes	
☐	☐	Demonstrate how to render a vaccine ineffective
☐	☐	Confer resistance to therapeutically useful antibiotics or antiviral agents
☐	☐	Enhance the virulence of a pathogen or render a nonpathogen virulent
☐	☐	Increase transmissibility of a pathogen
☐	☐	Alter the host range of a pathogen
☐	☐	Enable evasion of diagnostic/detection modalities
☐	☐	Enable the weaponization of a biological agent or toxin
☐	☐	Any other potentially harmful combination of experiments and agents

ChIP-seq

Data deposition

☐ Confirm that both raw and final processed data have been deposited in a public database such as GEO.

☐ Confirm that you have deposited or provided access to graph files (e.g. BED files) for the called peaks.

Data access links<br>*May remain private before publication.*	*For "Initial submission" or "Revised version" documents, provide reviewer access links. For your "Final submission" document, provide a link to the deposited data.*
Files in database submission	*Provide a list of all files available in the database submission.*

Genome browser session
(e.g. UCSC)

Provide a link to an anonymized genome browser session for "Initial submission" and "Revised version" documents only, to enable peer review. Write "no longer applicable" for "Final submission" documents.

Methodology

Replicates	*Describe the experimental replicates, specifying number, type and replicate agreement.*
Sequencing depth	*Describe the sequencing depth for each experiment, providing the total number of reads, uniquely mapped reads, length of reads and whether they were paired- or single-end.*
Antibodies	*Describe the antibodies used for the ChIP-seq experiments; as applicable, provide supplier name, catalog number, clone name, and lot number.*
Peak calling parameters	*Specify the command line program and parameters used for read mapping and peak calling, including the ChIP, control and index files used.*
Data quality	*Describe the methods used to ensure data quality in full detail, including how many peaks are at FDR 5% and above 5-fold enrichment.*
Software	*Describe the software used to collect and analyze the ChIP-seq data. For custom code that has been deposited into a community repository, provide accession details.*

Flow Cytometry

Plots

Confirm that:

☐ The axis labels state the marker and fluorochrome used (e.g. CD4-FITC).

☐ The axis scales are clearly visible. Include numbers along axes only for bottom left plot of group (a 'group' is an analysis of identical markers).

☐ All plots are contour plots with outliers or pseudocolor plots.

☐ A numerical value for number of cells or percentage (with statistics) is provided.

Methodology

Sample preparation	*Describe the sample preparation, detailing the biological source of the cells and any tissue processing steps used.*
Instrument	*Identify the instrument used for data collection, specifying make and model number.*
Software	*Describe the software used to collect and analyze the flow cytometry data. For custom code that has been deposited into a community repository, provide accession details.*
Cell population abundance	*Describe the abundance of the relevant cell populations within post-sort fractions, providing details on the purity of the samples and how it was determined.*
Gating strategy	*Describe the gating strategy used for all relevant experiments, specifying the preliminary FSC/SSC gates of the starting cell population, indicating where boundaries between "positive" and "negative" staining cell populations are defined.*

☐ Tick this box to confirm that a figure exemplifying the gating strategy is provided in the Supplementary Information.

Magnetic resonance imaging

Experimental design

Design type	*Indicate task or resting state; event-related or block design.*
Design specifications	*Specify the number of blocks, trials or experimental units per session and/or subject, and specify the length of each trial or block (if trials are blocked) and interval between trials.*
Behavioral performance measures	*State number and/or type of variables recorded (e.g. correct button press, response time) and what statistics were used to establish that the subjects were performing the task as expected (e.g. mean, range, and/or standard deviation across subjects).*

Acquisition

Imaging type(s) | *Specify: functional, structural, diffusion, perfusion.*

Field strength | *Specify in Tesla*

Sequence & imaging parameters | *Specify the pulse sequence type (gradient echo, spin echo, etc.), imaging type (EPI, spiral, etc.), field of view, matrix size, slice thickness, orientation and TE/TR/flip angle.*

Area of acquisition | *State whether a whole brain scan was used OR define the area of acquisition, describing how the region was determined.*

Diffusion MRI ☐ Used ☐ Not used

Preprocessing

Preprocessing software | *Provide detail on software version and revision number and on specific parameters (model/functions, brain extraction, segmentation, smoothing kernel size, etc.).*

Normalization | *If data were normalized/standardized, describe the approach(es): specify linear or non-linear and define image types used for transformation OR indicate that data were not normalized and explain rationale for lack of normalization.*

Normalization template | *Describe the template used for normalization/transformation, specifying subject space or group standardized space (e.g. original Talairach, MNI305, ICBM152) OR indicate that the data were not normalized.*

Noise and artifact removal | *Describe your procedure(s) for artifact and structured noise removal, specifying motion parameters, tissue signals and physiological signals (heart rate, respiration).*

Volume censoring | *Define your software and/or method and criteria for volume censoring, and state the extent of such censoring.*

Statistical modeling & inference

Model type and settings | *Specify type (mass univariate, multivariate, RSA, predictive, etc.) and describe essential details of the model at the first and second levels (e.g. fixed, random or mixed effects; drift or auto-correlation).*

Effect(s) tested | *Define precise effect in terms of the task or stimulus conditions instead of psychological concepts and indicate whether ANOVA or factorial designs were used.*

Specify type of analysis: ☐ Whole brain ☐ ROI-based ☐ Both

Statistic type for inference (See Eklund et al. 2016) | *Specify voxel-wise or cluster-wise and report all relevant parameters for cluster-wise methods.*

Correction | *Describe the type of correction and how it is obtained for multiple comparisons (e.g. FWE, FDR, permutation or Monte Carlo).*

Models & analysis

n/a	Involved in the study
☐	☐ Functional and/or effective connectivity
☐	☐ Graph analysis
☐	☐ Multivariate modeling or predictive analysis

Functional and/or effective connectivity | *Report the measures of dependence used and the model details (e.g. Pearson correlation, partial correlation, mutual information).*

Graph analysis | *Report the dependent variable and connectivity measure, specifying weighted graph or binarized graph, subject- or group-level, and the global and/or node summaries used (e.g. clustering coefficient, efficiency, etc.).*

Multivariate modeling and predictive analysis | *Specify independent variables, features extraction and dimension reduction, model, training and evaluation metrics.*

March 2021
