# Supplementary material for: Elevated extracellular matrix protein 1 in circulating extracellular vesicles supports breast cancer progression under obesity conditions
Source: Nat Commun. 2024 Feb 24;15:1685. doi: 10.1038/s41467-024-45995-5 (PMC10894219; doi:10.1038/s41467-024-45995-5)
Supplement: Supplementary file 4 — Description of Additional Supplementary File [file 41467_2024_45995_MOESM4_ESM.docx]

**Supplementary Data 1**

The proteomics data showing the differential expressed proteins (DEPs) in the circulating sEVs of obese or overweight and healthy normal weight human subjects.

**Supplementary Data 2**

The proteomics data showing the differential expressed proteins (DEPs) in the circulating sEVs of control diet and high-fat diet-induced obesity mouse models.

**Supplementary Data 3**

The selected peptides for the quantification for multiple reaction monitoring (MRM) mass spectrometry.

**Supplementary Data 4**

The selected proteins in the circulating sEVs of obese or overweight and healthy normal weight human subjects examined by MRM-mass spectrometry. Two-sided unpaired *t*-test for statistical analysis.

**Supplementary Data 5**

Optimization parameters of Degrouping voltage (DP) and collision energy (CE)
